# Phosphorylation of different tau sites during progression of Alzheimer’s disease

**DOI:** 10.1186/s40478-018-0557-6

**Published:** 2018-06-29

**Authors:** Joerg Neddens, Magdalena Temmel, Stefanie Flunkert, Bianca Kerschbaumer, Christina Hoeller, Tina Loeffler, Vera Niederkofler, Guenther Daum, Johannes Attems, Birgit Hutter-Paier

**Affiliations:** 1 QPS Austria GmbH, Neuropharmacology, Parkring 12, 8074 Grambach, Austria; 2 Institute for Biochemistry, Graz University of Technology, Graz, Austria; 3 Institute of Zoology, Karl Franzens University, Graz, Austria; 4 Institute of Neuroscience and Newcastle University Institute for Ageing Campus for Ageing and Vitality, Newcastle University, Newcastle upon Tyne, NE4 5PL UK

**Keywords:** Microtubule-associated protein tau, Phosphorylation, Cingulate, Frontal, Occipital and temporal cortex, Transentorhinal region, Immunofluorescent labeling

## Abstract

Alzheimer’s disease is characterized by accumulation of amyloid plaques and tau aggregates in several cortical brain regions. Tau phosphorylation causes formation of neurofibrillary tangles and neuropil threads. Phosphorylation at tau Ser202/Thr205 is well characterized since labeling of this site is used to assign Braak stage based on occurrence of neurofibrillary tangles. Only little is known about the spatial and temporal phosphorylation profile of other phosphorylated tau (ptau) sites. Here, we investigate total tau and ptau at residues Tyr18, Ser199, Ser202/Thr205, Thr231, Ser262, Ser396, Ser422 as well as amyloid-β plaques in human brain tissue of AD patients and controls. Allo- and isocortical brain regions were evaluated applying rater-independent automated quantification based on digital image analysis. We found that the level of ptau at several residues, like Ser199, Ser202/Thr205, and Ser422 was similar in healthy controls and Braak stages I to IV but was increased in Braak stage V/VI throughout the entire isocortex and transentorhinal cortex. Quantification of ThioS-stained plaques showed a similar pattern. Only tau phosphorylation at Tyr18 and Thr231 was already significantly increased in the transentorhinal region at Braak stage III/IV and hence showed a progressive increase with increasing Braak stages. Additionally, the increase in phosphorylation relative to controls was highest at Tyr18, Thr231 and Ser199. By contrast, Ser396 tau and Ser262 tau showed only a weak phosphorylation in all analyzed brain regions and only minor progression. Our results suggest that the ptau burden in the isocortex is comparable between all analyzed ptau sites when using a quantitative approach while levels of ptau at Tyr18 or Thr231 in the transentorhinal region are different between all Braak stages. Hence these sites could be crucial in the pathogenesis of AD already at early stages and therefore represent putative novel therapeutic targets.

**Electronic supplementary material:**

The online version of this article (10.1186/s40478-018-0557-6) contains supplementary material, which is available to authorized users.

## Introduction

Alzheimer’s disease (AD) is neuropathologically characterized by two hallmark lesions, which are extracellular amyloid-β (Aβ) plaques and intracellular accumulations of abnormally phosphorylated tau. Aβ plaques initially develop in neocortical regions and then progress to the limbic system, subcortical nuclei and reach the cerebellum at late stages of the disease [41]. Tau pathology manifests as neurofibrillary tangles (NFTs) and neuropil threads (NTs) and primarily accumulates in the entorhinal region and subsequently progresses to the limbic system and neocortical regions as reflected by NFT Braak stages [8]. Tau aggregation depends on several posttranslational modifications, including but not limited to, truncation, acetylation, ubiquitination, sumoylation and phosphorylation [13, 29, 34]. The best analyzed posttranslational modification in AD is abnormal phosphorylation of tau which in AD is referred to as hyperphosphorylation and that is characterized by an at least 3-fold increase of tau phosphorylation relative to controls. Over 70 potential tau phosphorylation (ptau) sites spanning almost the entire protein structure and including some phosphorylation sites are assumed to be pathologically relevant [40]. Some of these ptau sites are known to be abnormally phosphorylated in paired helical filaments (PHFs), NFTs or NTs during progression of AD but are not phosphorylated in healthy brains [10, 15, 22, 26, 28]. Several of these ptau sites are also phosphorylated in the fetal brain and are thus associated with embryonic brain developmental processes [10, 15, 22]. In the diseased brain, ptau sites are commonly associated with tau aggregation processes such as incomplete binding and destabilization of microtubules, causing the transition from pre-tangles to NFT formation [12, 16, 21, 28, 32].

The temporal and spatial phosphorylation pattern of tau residue Ser202/Thr205 has already been well characterized since staging of AD cases is based on labeling of ptau Ser202/Thr205 using the specific antibody AT8 [1, 9, 20, 46]. However, the phosphorylation profile of other ptau sites known to be involved in AD pathology is so far strongly neglected and analyzed only by ELISA and dot blots of AD brain tissue [20, 46].

Phosphorylation at several residues during disease progression has been repeatedly investigated in tau animal models but barely in AD brain tissue [4, 7, 14, 20, 46]. Hence, the present study aims to evaluate the spatial and temporal phosphorylation profile of different tau sites by applying rater-independent automated quantification based on digital image analysis. We therefore quantified the expression of total tau, amyloid-β plaques and the phosphorylation of seven different tau sites (Tyr18, Ser199, Ser202/Thr205, Thr231, Ser262, Ser396, Ser422) in iso- and allocortical brain regions of AD cases and controls.

## Materials and methods

### Human brain samples

Paraffin sections of 6 μm thickness and frozen samples from AD patients of three different disease stages [Braak stages I/II, III/IV and *V*/VI; [9]] and control individuals with Braak stage 0 were provided by the Newcastle Brain Tissue Resource (NBTR), Newcastle University, UK in accordance with the approval of the joint Ethics Committee of Newcastle and North Tyneside Health Authority and following NBTR brain banking procedures. The neuropathological diagnosis was performed according to internationally accepted criteria [31] (Table 1). Tissue of one patient of Braak stage V/VI yielded clearly stronger pathology compared to the other cases, representing an overshoot outlier. Data of this patient were excluded for pThr231 calculations. For histological analyses, five cases per group were investigated using sections of 5 brain regions per individual: temporal cortex (TeCtx), frontal cortex (FrCtx), cingulate cortex (CiCtx), occipital cortex (OcCtx), and transentorhinal region (TEntR). All histological assessments were done blinded to clinical and neuropathological data.

**Table 1:** Individual case information. Demographic patient information

	Case	Age	Sex	Braakstage	Post mortem delay to fixation in hours	Fixation time in weeks	Clinical information	# on Western blot
Controls	1	68	M	0	54	7	Cognitively normal	1
	2	55	M	0	41	11	Cognitively normal	2
	3	70	M	0	72	6	Cognitively normal	–
	4	78	F	0	34	8	Cognitively normal	4
	5	73	M	0	25	9	Cognitively normal	–
	mean	68.8		0	45.2	8.2		
Braak I/II Low	6	96	F	2	114	49	Mild dementia	6
	7	77	M	2	83	15	Cognitively normal	7
	8	94	F	2	15	9	Cognitively normal	–
	9	70	M	2	39	7	Multiple psychiatric and physical problems	9
	10	74	F	1	49	10	Cognitively normal	10
	mean	82.2		1.8	60	18		
Braak III/IV Mod.	11	75	M	4	82	23	Cognitively normal	11
	12	79	M	3	13	15	Cognitively normal	12
	13	81	M	3	82	8	Unspecified dementia	13
	14	98	F	3	59	8	Cognitively normal	14
	15	91	M	3	48	9	Moderate cognitive impairment and vascular disease	15
	mean	84.8		3.2	56.8	12.6		
Braak V/VI High	16	84	F	6	47	16	Severe dementia, anxiety and depression	–
	17	77	F	6	63	5	Dementia	17
	18	80	F	6	32	16	Dementia	18
	19	86	F	6	5	6	Dementia	19
	20	89	F	6	85	8	Dementia	–
	mean	83.2		6	46.4	10.2		

### Immunofluorescent labeling

Sections were deparaffinized for 10 min in Tissue Clear (Sakura, 1466, Netherlands) and 5 min in Tissue Clear/100% ethanol, washed for 5 min in 100% ethanol and then subsequently rehydrated with decreasing alcohol concentrations (96, 70 and 50% ethanol for 2 min each). Thereafter, sections were washed twice for 5 min in PBS. Sections for labeling of human total tau, ThioflavinS (ThioS), pThr231 tau, pSer199 tau, pTyr18 tau and pSer396 tau were treated for 15 min with citrate buffer (Thermo Scientific, AP-9003) at 95 °C in a steamer and cooled down to room temperature (RT) for another 15 min. For ThioS staining, sections were then labeled with 0.5% ThioS (Sigma-Aldrich, T1892) for 7 min under light protection. Afterwards, sections were pretreated with ice-cold sodium borohydride/PBS solution (1 mg/ml; Sigma-Aldrich, 213,462) for 4 min and tissue was then permeabilized with 1% Triton® X-100/PBS (AppliChem, A1388) for 10 min. Non-specific labeling was blocked by incubating sections for 30–60 min either with 10% donkey serum/PBS or 10% horse serum/PBS. For antigen detection, sections were incubated with primary antibodies (Table 2) in a damp chamber. Primary antibody binding was visualized by incubating sections with secondary fluorophore conjugated antibodies (Table 3) for 60 min. Furthermore, cell nuclei of tissue labeled for pSer199 tau, pSer396 tau and pSer422 tau were visualized by counterstaining with 4′,6-Diamidin-2-phenylindol-working solution (DAPI, AppliChem, A1001) for 15 min, differentiated with 70% ethanol and washed for 5 min in PBS. Finally, sections were washed in ddH_2_O and covered with Moviol and coverslips.

**Table 2:** List of primary antibodies for histology

Antigen	Clone	Species	Source	Order #	Dilution	Incubation time
Total human tau	HT7	mouse	Pierce Biotechnology, USA	MN1000	1:500	overnight at 4 °C
pThr231	AT180	mouse	Pierce Biotechnology, USA	MN1040	1:200	overnight at 4 °C
pSer202 + pThr205	AT8	mouse	Thermo Scientific, USA	MN1020	1:200	overnight at 4 °C
pSer199	polyclonal	rabbit	Invitrogen Corporation, USA	44-734G	1:500	1 h at RT
pTyr18	9G3	mouse	MédiMabs, Canada	MM-0194-P	1:1000	1 h at RT
pSer396	PHF13	mouse	Cell Signaling Technology, USA	9632	1:500	1 h at RT
pSer262	polyclonal	rabbit	MBL International, USA	AT-5020	1:250	overnight at 4 °C
pSer422	5.6.11	mouse	Roche Diagnostic, Switzerland	N/A	1:800	overnight at 4 °C

**Table 3:** List of secondary antibodies

Antibody	Conjugation	Source	Order #	To visualize
Donkey Anti-Mouse IgG H&L	AlexaFluor 555	abcam, UK	ab150110	pSer422 tau
				pSer396 tau
				pSer202/ pThr205 tau
Donkey Anti-Rabbit IgG H&L	DyLight 650	abcam, UK	ab96922	pSer199 tau
				pSer262 tau
Donkey Anti-Mouse IgG H&L	DyLight 550	abcam, UK	ab98795	pThr231 tau
Donkey Anti-Mouse IgG (H + L)	Cyanine Cy3	Jackson ImmunoResearch, USA	715–165-151	Total human tau
Donkey Anti-Mouse IgG H&L	DyLight 650	abcam, UK	ab98797	pTyr18 tau
Dilution of all secondary antibodies was 1:500				

In order to use the human sections efficiently, double labelings were performed: pSer202/Thr205 combined with pSer262 (Additional file 1: Online Source 9); ThioS combined with HT7; pSer199 combined with pSer396. Labelings against pTyr18, pThr231 and pSer422 were performed without co-labeling of another ptau residue.

### Imaging and image analysis

Of each labeled brain section two mosaic images with a size of approximately 3 mm^2^ including white and grey matter were captured at different z-levels and finally projected to 2D.

Imaging of immunofluorescent labelings of pSer202/Thr205 tau, pSer199 tau, pSer396 tau, pSer262 tau and pSer422 tau was performed using a Nikon Eclipse E400 microscope with a high aperture lens (20× lens, numerical aperture 0.5, 1× optocoupler), equipped with a Nikon DS-Qi1MC camera and controlled by the Nikon NIS-Elements AR software. For quantification of total human tau, pThr231 tau, pTyr18 tau and ThioS images were recorded using a Zeiss AxioImager.Z1 microscope with a high aperture lens and an AxioVision 4.8 software-driven AxioCam MRm digital camera (20× lens, numeric aperture 0.8, 1× optocoupler).

Quantitative image analysis was performed using Image-Pro Plus (version 6.2, Media Cybernetics, Inc., Rockville, USA). Grey-scale single channel images were corrected for background intensities using lowpass filtering, and signal from autofluorescent objects (mostly lipofuscin and erythrocytes) was subtracted from the channel used for immunofluorescent labeling. Objects were identified by a combination of Edge+ filter, adequate thresholding, and size and shape restrictions (Additional file 1: Online Source 10). After defining the parameters for detecting the targeted objects, macro-driven quantitative image analysis ran automatically using the same parameters on all images. The quantitative results are therefore unbiased, rater-independent and fully reproducible.

### Preparation of sarcosyl insoluble fraction from frozen samples

Frozen transentorhinal cortex tissue was homogenized in 9 μl per mg of cold extraction buffer 1 (25 mM Tris HCl pH = 7.4, 150 mM NaCl, 1 mM EDTA, 1 mM EGTA, 10 mM ß-glycerophosphate, 30 mM NaF, 2 mM Na3VO4, protease and phosphatase inhibitor cocktails) and centrifuged at 80,000 g for 15 min at 4 °C. The pellet was resuspended in extraction buffer 2 (10 mM Tris HCl pH = 7.4, 800 mM NaCl, 300 mM sucrose, 1 mM EGTA, protease and phosphatase inhibitor cocktails) and centrifuged at 4000 g for 10 min at 4 °C. The Supernatant was transferred to a fresh tube and sarcosyl (30% aqueous solution) was added to a final concentration of 1% and incubated for 1.5 h at room temperature. After centrifugation at 80,000 g for 30 min at 4 °C, the supernatant was discarded and the pellet resuspended in buffer 3 (50 mM Tris-HCl, pH = 7.4). The suspension was aliquoted and frozen until used for Western blotting.

### Western blot analyses

Equal amounts of proteins of each brain extract were separated by molecular weight on a SDS-PAGE polyacrylamide gel. A protein marker visualized correct separation of the proteins and confirmed the correct protein band size. Subsequently, proteins were transferred onto a 0.45 μm nitrocellulose membrane using a wet blot chamber (Bio-Rad, Hercules, USA), blocked with 5% non-fat dry milk in 1 × TBS and incubated with primary antibodies overnight. To blot for total tau, pTyr18, and pSer199 the same primary antibodies as used for histological labeling were used in a concentration of 1:1000 (Table 2). Antibodies AT180 and AT8 to label pThr231 and pSer202/Thr205, respectively, did not give any signal in the Western blot and were thus substituted by a rabbit polyclonal antibody against pThr231 (1:1000; Signalway, SAB1110–2, College Park, USA) and a rabbit monoclonal antibody, clone EPR2402, against pSer202/Thr205 (1:3000; abcam, ab108387, Cambridge, UK). All primary antibodies were incubated for 2 h at room temperature. Afterwards, membranes were washed in TBS and incubated in horseradish peroxidase-coupled secondary antibodies for 1 h at room temperature (1:5000; donkey-anti-rabbit IgG: NA934/ GENA934; sheep-anti-mouse IgG: NXA931; GE-Healthcare, Little Chalfont, UK). Proteins were detected using Wester-Bright ECL spray (Advansta, Menlo Park, USA) and placed on a X-ray film. As loading control a rabbit polyclonal GAPDH antibody (Sigma-Aldrich, G9545, St. Louis, USA) was co-labeled on each blot. To blot with 6 different antibodies, three blots were used to prevent false signals due to stripping, reblotting or blocking of antibody binding by previously used antibody. For each of the three blots, GAPDH as loading control is shown. Densitometry of all bands was performed using Image J software. Cases 3, 5, 8, 16 and 20 according to Table 1 are missing in this analysis since no frozen tissue was available from the corresponding brain region.

### Statistical analyses

All statistical analyses and preparation of graphs were conducted using Graph-Pad Prism (version 4.03, San Diego, CA, USA). Descriptive statistical analyses were performed on all evaluated parameters including the evaluation of normal distribution using the Kolmogorov-Smirnov test.

Group variances were calculated either by one-way or two-way ANOVA. If a significant interaction among groups was detected, Newman-Keuls or Bonferroni’s *post-hoc* analysis was followed. A detailed description of performed statistical analyses is given in the appropriate figure legend. Data were averaged and represented as mean + standard error of mean (SEM). An α-error level of *p* < 0.05 was considered significant.

## Results

**Fig. 1:**
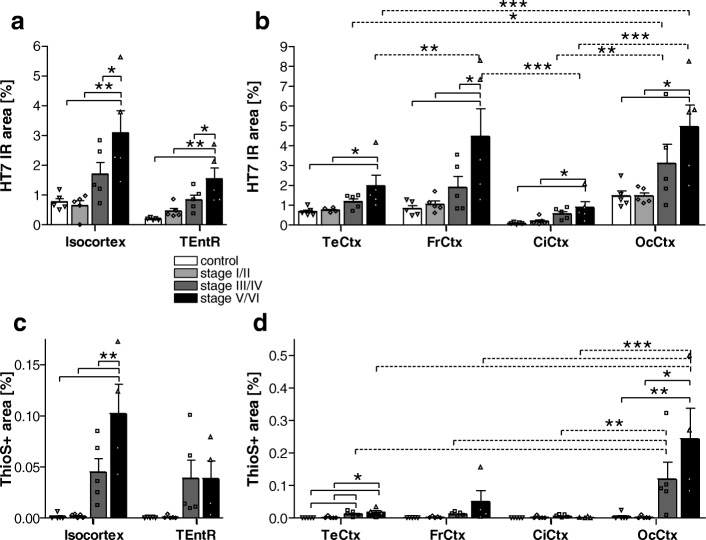
Quantification of total human tau and plaque load in the cortex of AD cases. **a** Total human tau (HT7 antibody) immunoreactive area in percent in the isocortex and TEntR (allocortex) of different AD stages. **c** β sheet (ThioflavinS staining) positive area in percent in the isocortex and TEntR of different AD stages. Isocortical data in **a** and **c** present mean of different cortical regions that are shown separately in **b** and **d**, respectively. Two way ANOVA followed by Bonferroni’s *posthoc* test. Mean + SEM; *N* = 5.*p < 0.05; **p < 0.01; ***p < 0.001. **c**, **d** One outlier of Braak stage V/VI excluded for all ThioS labelings. Solid lines: Comparison of AD stages within a brain region; dotted lines: comparison of brain regions of the same AD Braak stage. CiCtx: cingulate cortex; FrCtx: frontal cortex; OcCtx: occipital cortex; TeCtx: temporal cortex; TEntR: transentorhinal region

In order to evaluate the overall human tau expression levels, brain tissues were labeled with the anti-human total tau HT7 antibody. Quantification of the HT7 immunoreactive area in the isocortex and transentorhinal region (TEntR) revealed a progressive increase of total tau with advancing Braak stages. This increase in total tau signal was significant at Braak stage V/VI compared to healthy controls but also compared to both lower Braak stages I/II and III/IV (Fig. 1a). Data of the isocortex in Fig. 1a are the mean of four distinct isocortical regions, namely temporal, frontal, cingulate and occipital cortex. Separate presentation of these brain regions shows that total tau expression levels were very low in the cingulate cortex but relatively high in the occipital cortex of healthy controls (Fig. 1b). In all isocortical regions total tau levels started to increase at Braak stage III/IV and were significantly higher at Braak stage V/VI compared to controls or Braak stage I/II. At Braak stage V/VI total tau levels were highest in the frontal and occipital cortex (Fig. 1b). Since amyloid-β plaques are, next to tau, a major pathological hallmark of AD, the ThioflavinS (ThioS) stained area was quantified in the isocortex and TEntR of AD patients and healthy controls (Fig. 1c). Threshold-based identification of ThioS staining combined with a size restriction filter enabled automatic quantification of plaque cores while excluding NFTs (Additional file 1: Online Source 11). We found that ThioS signal was significantly increased in the isocortex of Braak stage V/VI patients compared to controls and all other Braak stages; a first non-significant increase in the isocortical ThioS signal could be observed at Braak stage III/IV (Fig. 1c). In the TEntR, ThioS signal slightly increased at Braak stage III/IV but did not further increase at Braak V/VI. Almost no ThioS signal could be observed in the cingulate cortex. In the temporal and frontal cortex the ThioS signal was very low while only in the occipital cortex the ThioS signal was well measurable and progressively increasing starting at Braak stage III/IV (Fig. 1d).

After evaluating the overall total tau and plaque load, we measured the phosphorylation levels of ptau at seven different residues. First, we show phosphorylation of pSer202/Thr205 tau since this phosphorylation site is already well characterized and thus can be utilized to validate the results.

### pSer202/Thr205 tau

**Fig. 2:**
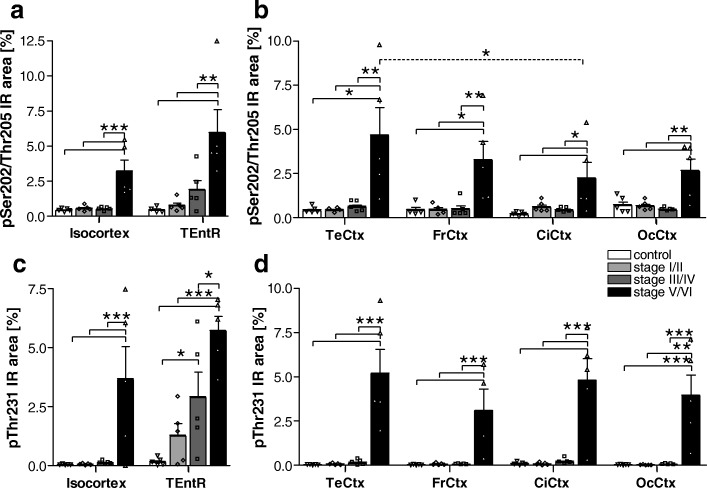
Quantification of tau phosphorylation at pSer202/Thr205 and pThr231 in the cortex of AD cases. **a** Human pSer202/Thr205 tau immunoreactive area in percent in the isocortex and TEntR (allocortex) of different AD stages. **c** Human pThr231 tau immunoreactive area in percent in the isocortex and TEntR of different AD stages. Isocortical data in **a** and **c** present mean of different cortical regions that are shown separately in **b** and **d**, respectively. Two way ANOVA followed by Bonferroni’s *posthoc* test. Mean + SEM; *N* = 5.*p < 0.05; **p < 0.01; ***p < 0.001. Solid lines: Comparison of AD stages within a brain region; dotted lines: comparison of brain regions of the same AD Braak stage. CiCtx: cingulate cortex; FrCtx: frontal cortex; OcCtx: occipital cortex; TeCtx: temporal cortex; TEntR: transentorhinal region

Phosphorylation of tau at Ser202/Thr205 was low in all isocortical brain regions up to Braak stage III/IV. Only at Braak stage V/VI Ser202/Thr205 tau phosphorylation significantly increased about 4- to 13-fold above control levels (Fig. 2a, b). At Braak stage V/VI the highest Ser202/Thr205 tau phosphorylation could be observed in the temporal cortex while the lowest phosphorylation was in the cingulate cortex (Fig. 2b). Analysis of the allocortical TEntR showed a first but not significant pSer202/Thr205 tau increase in Braak stage III/IV that became significant at Braak stage V/VI (Fig. 2a).

### pThr231 tau

Phosphorylation of tau at Thr231 was also low in all isocortical brain regions up to Braak stage III/IV. Only at Braak stage V/VI Thr231 tau phosphorylation significantly increased about 30- to 160-fold above control levels (Fig. 2c, d). At Braak stage V/VI the highest Thr231 tau phosphorylation could be observed in the temporal cortex while the lowest phosphorylation was in the frontal cortex (Fig. 2d). Analysis of the allocortical TEntR showed a first but not significant pThr231 tau increase at Braak stage I/II that became significant compared to healthy controls at Braak stage III/IV and further significantly increased at Braak stage V/VI compared to all other groups (Fig. 2c).

### pSer199 tau

**Fig. 3:**
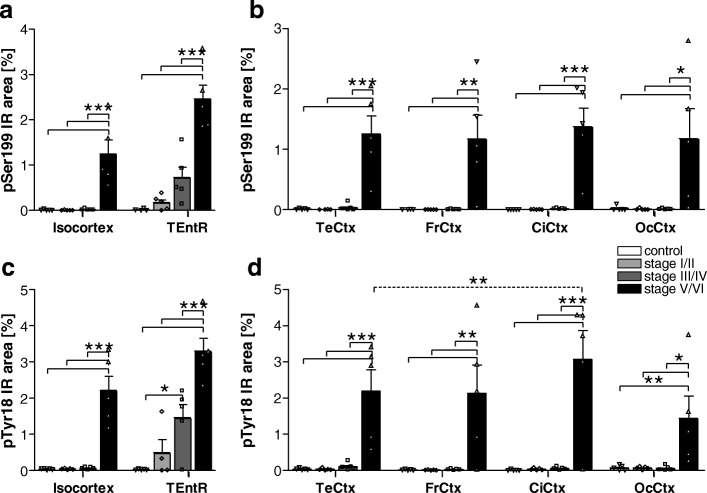
Quantification of tau phosphorylation at pSer199 and pTyr18 in the cortex of AD cases. **a** Human pSer199 tau immunoreactive area in percent in the isocortex and TENtR (allocortex) of different AD stages. **c** Human pTyr18 tau immunoreactive area in percent in the isocortex and TEntR of different AD stages. Isocortical data in **a** and **c** present mean of different cortical regions that are shown separately in **b** and **d**, respectively. Two way ANOVA followed by Bonferroni’s *posthoc* test. Mean + SEM; *N* = 5.*p < 0.05; **p < 0.01; ***p < 0.001. Solid lines: Comparison of AD stages within a brain region; dotted lines: comparison of brain regions of the same AD Braak stage. CiCtx: cingulate cortex; FrCtx: frontal cortex; OcCtx: occipital cortex; TeCtx: temporal cortex; TEntR: transentorhinal region

Overall, phosphorylation of tau at Ser199 was low compared to other phosphorylation sites. In all isocortical regions Ser199 tau phosphorylation started late, namely at Braak stage V/VI (Fig. 3a, b). At Braak stage V/VI Ser199 tau phosphorylation was at comparable levels in all isocortical regions and about 50- (occipital cortex) to 1300-fold (cingulate cortex) increased compared to healthy controls (Fig. 3b). Analysis of the allocortical TEntR showed a progressive increase in Ser199 tau phosphorylation starting at Braak stage I/II but differences between Braak stages became significant only at stage V/IV and were then about 3.5-fold compared to Braak stage III/IV and 160-fold compared to healthy controls (Fig. 3a). Phosphorylation of Ser199 tau in the TEntR of late Braak stages was higher compared to all isocortical regions.

### pTyr18 tau

The phosphorylation profile of pTyr18 tau in the isocortex and TEntR was very similar to pSer199 tau although the final phosphorylation levels in Braak stage V/VI were slightly higher and increased about 20- to 140-fold above control levels (Fig. 3c, d). The progression in the TEntR was already significant at Braak stage III/IV compared to stage I/II and controls. At Braak stage V/VI the highest pTyr18 tau could be observed in the TEntR and cingulate cortex while the lowest phosphorylation was measured in the occipital cortex (Fig. 3c, d).

### pSer396 tau

**Fig. 4:**
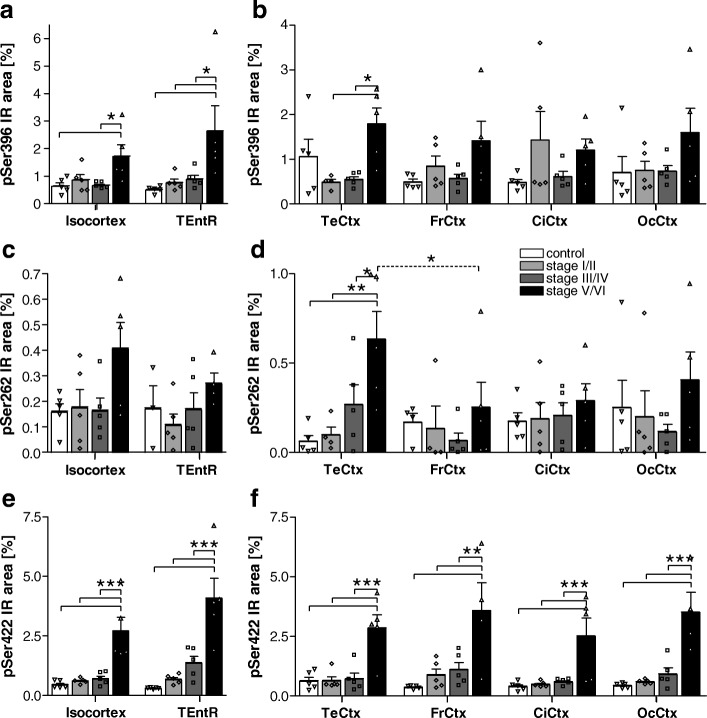
Quantification of tau phosphorylation at pSer396, pSer262 and pSer422 in the cortex of AD cases. **a** Human pSer396 tau immunoreactive area in percent in the isocortex and TEntR (allocortex) of different AD stages. **c** Human pSer422 tau immunoreactive area in percent in the isocortex and TEntR of different AD stages. **e** Human pSer262 tau immunoreactive area in percent in the isocortex and TEntR of different AD stages. Isocortical data in **a**, **c** and **e** present mean of different cortical regions that are shown separately in **b**, **d** and **f**, respectively. Two way ANOVA followed by Bonferroni’s *posthoc* test. Mean + SEM; *N* = 5.*p < 0.05; **p < 0.01; ***p < 0.001. Solid lines: Comparison of AD stages within a brain region; dotted lines: comparison of brain regions of the same AD Braak stage. CiCtx: cingulate cortex; FrCtx: frontal cortex; OcCtx: occipital cortex; TeCtx: temporal cortex; TEntR: transentorhinal region

Phosphorylation of tau at Ser396 shows higher variability than other ptau sites. In the isocortex overall Ser396 tau phosphorylation starts very late in Braak stage V/VI (Fig. 4b). When analyzing different isocortical regions separately, it seemed that pSer396 tau was slightly increased in the temporal cortex of healthy controls and in the cingulate cortex at early Braak stage I/II but these variations were not significant (Fig. 4b). Otherwise, Ser396 tau phosphorylation in all investigated regions only increased about 2- to 5-fold at Braak stage V/VI compared to control levels. Due to the high inter-individual variation this difference was only significant in the temporal cortex compared to Braak stage I/II and III/IV (Fig. 4b). In the allocortical TEntR pSer396 tau increased significantly at Braak stage V/VI compared to all other groups (Fig. 4a).

### pSer262 tau

Phosphorylation of tau at Ser262 was very low and showed almost no progression in higher Braak stages (Fig. 4c). Only in the TEntR a significant 8-fold increase could be noted between Braak stage I/II and V/VI (Fig. 4d).

### pSer422 tau

The phosphorylation profile of tau at Ser422 was comparable in all isocortical regions and also the allocortical TEntR. In all analyzed regions phosphorylation significantly increased at Braak stage V/VI (Fig. 4e, f) and was about 3- to 13-fold higher compared to the control group.

### Relative signal increase of ThioS and ptau

**Fig. 5:**
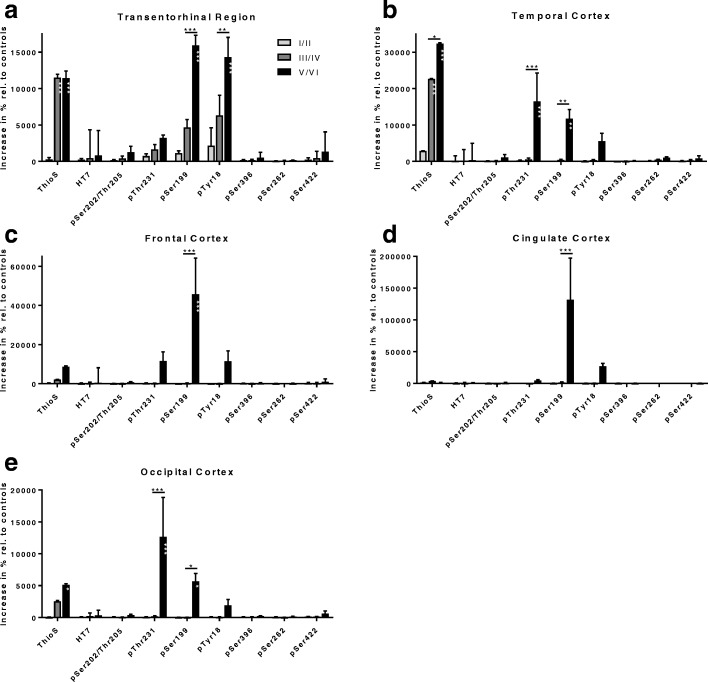
Signal increase relative to control brain tissue in the cortex of AD cases. Values are calculated from means of absolute IR area from Figs. 1, 2, 3 and 4 relative to control brain samples. Calculation is shown for ThioS, HT7, pSer202/Thr205, pThr231, pSer199, pTyr18, pSer396, pSer262 and pSer422 in the transentorhinal (**a**), temporal (**b**), frontal (**c**), cingulate (**d**) and occipital cortex (**e**) at Braak stages I/II; III/IV and V/VI. Two way ANOVA followed by Bonferroni’s *posthoc* test. Mean + SEM. White asterisks within bars mark significance compared to Braak stage I/II. *p < 0.05; **p < 0.01; ***p < 0.001

When evaluating the signal increase relative to healthy control brain tissue, it became evident that ThioS labeling in the TEntR increased already at Braak stage III/IV and reached a plateau at Braak stages III/IV and V/VI. The phosphorylation at tau Tyr18 and tau Ser199 severely increased at the same time at Braak stage V/VI with already a slight increase at Braak stage III/IV (Fig. 5a). Compared to this tau phosphorylation the changes at other sites were only minor. Analysis of the phosphorylation relative to healthy control tissue in isocortical brain regions (Fig. 5b-e) showed a progressive increase of ThioS labeling in all regions with highest values in the temporal cortex (Fig. 5b). Additionally, tau phosphorylation was severely increased at Thr231, Ser199 and Tyr18. Tau phosphorylation at Thr231 was highest in the temporal cortex (Fig. 5b) while phosphorylation at Ser199 and Tyr18 was highest in the frontal cortex (Fig. 5c). Also in the isocortex tau phosphorylation changes at other sites are only minor compared to Thr231, Ser199 and Tyr18.

**Fig. 6:**
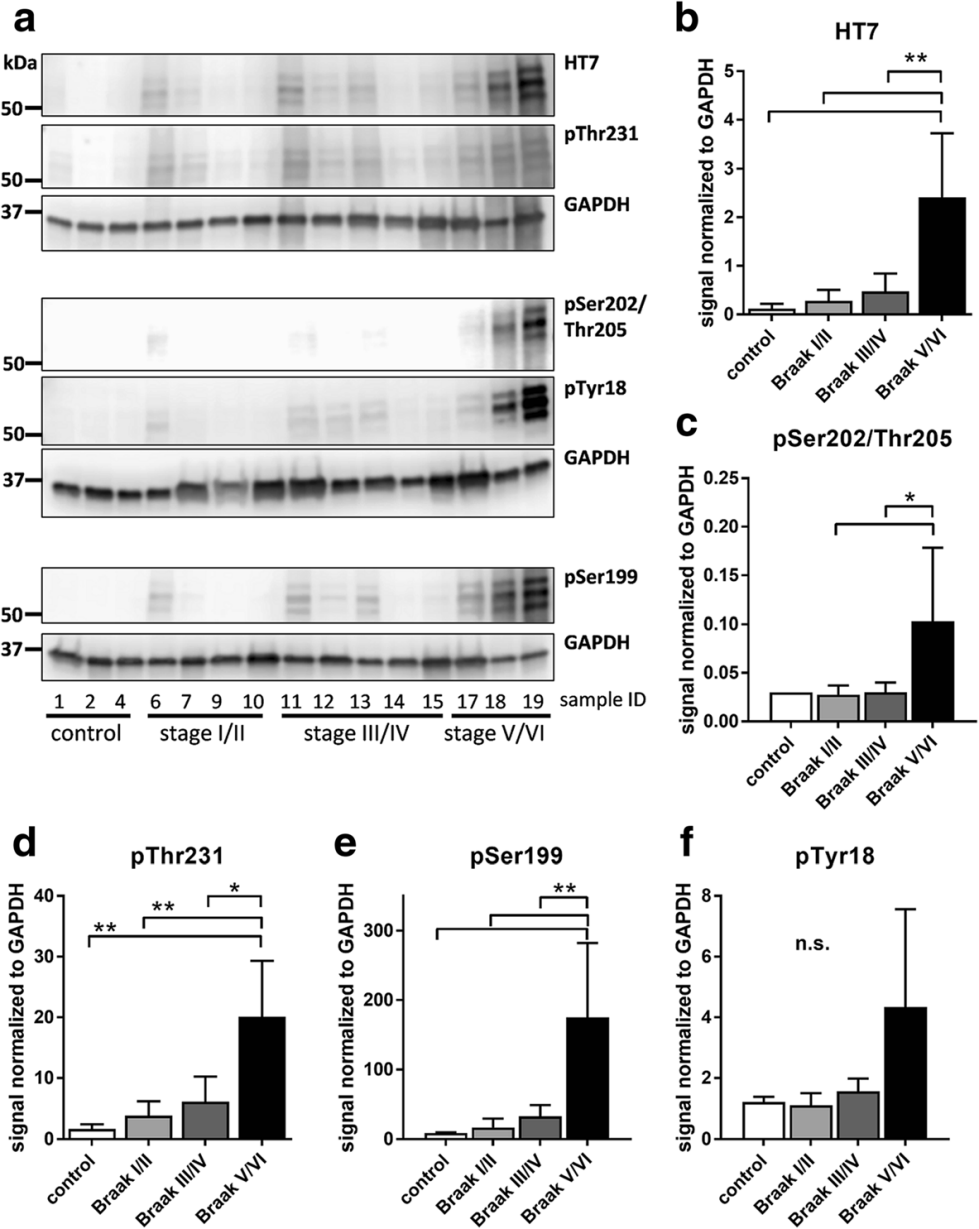
Quantification of total tau and tau phosphorylation in the transentorhinal cortex of AD cases by Western blotting. **a**: Western blots of total tau, pThr231, pSer202/Thr205, pTyr18, and pSer199. GAPDH was used as loading control. Quantification of Western blot for (**b**) total tau by HT7 antibody, (**c**) pSer202/Thr205, (**d**) pThr231, (**e**) pSer199, and (**f**) pTyr18. All samples shown in (**a**) were used for quantification of (**b**-**f**). One way ANOVA followed by Tukey’s multiple comparisons test. Mean + SEM. *p < 0.05; **p < 0.01; n.s.: not significant

To validate results obtained by immunofluorescent labeling, Western blots of AD and control cases were performed (Fig. 6a). Cases showing an increase in total tau also showed a signal increase in all analyzed ptau residues. Furthermore, the majority of human cases with strong ptau signals were clinically diagnosed with mild dementia (case 6 and 12) or dementia (case 17, 18, 19, Table 1), suggesting a correlation between phosphorylation and cognitive status. Even that tissue of some cases was missing the quantification of Western blot results shows an overall conformity compared to immunofluorescent labelings. Total tau by HT7 labeling (Fig. 6b), pSer202/205 (Fig. 6c), and pSer199 (Fig. 6e) also showed a late signal increase in Braak stage V/VI with only weak signals in earlier Braak stages. Western blotting of pThr231 (Fig. 6d) and pTyr18 (Fig. 6f) also showed a late signal increase in Braak stage V/VI while signals of immunofluorescent labelings increased already in earlier Braak stages (Fig. 2c and Fig. 3c).

**Fig. 7:**
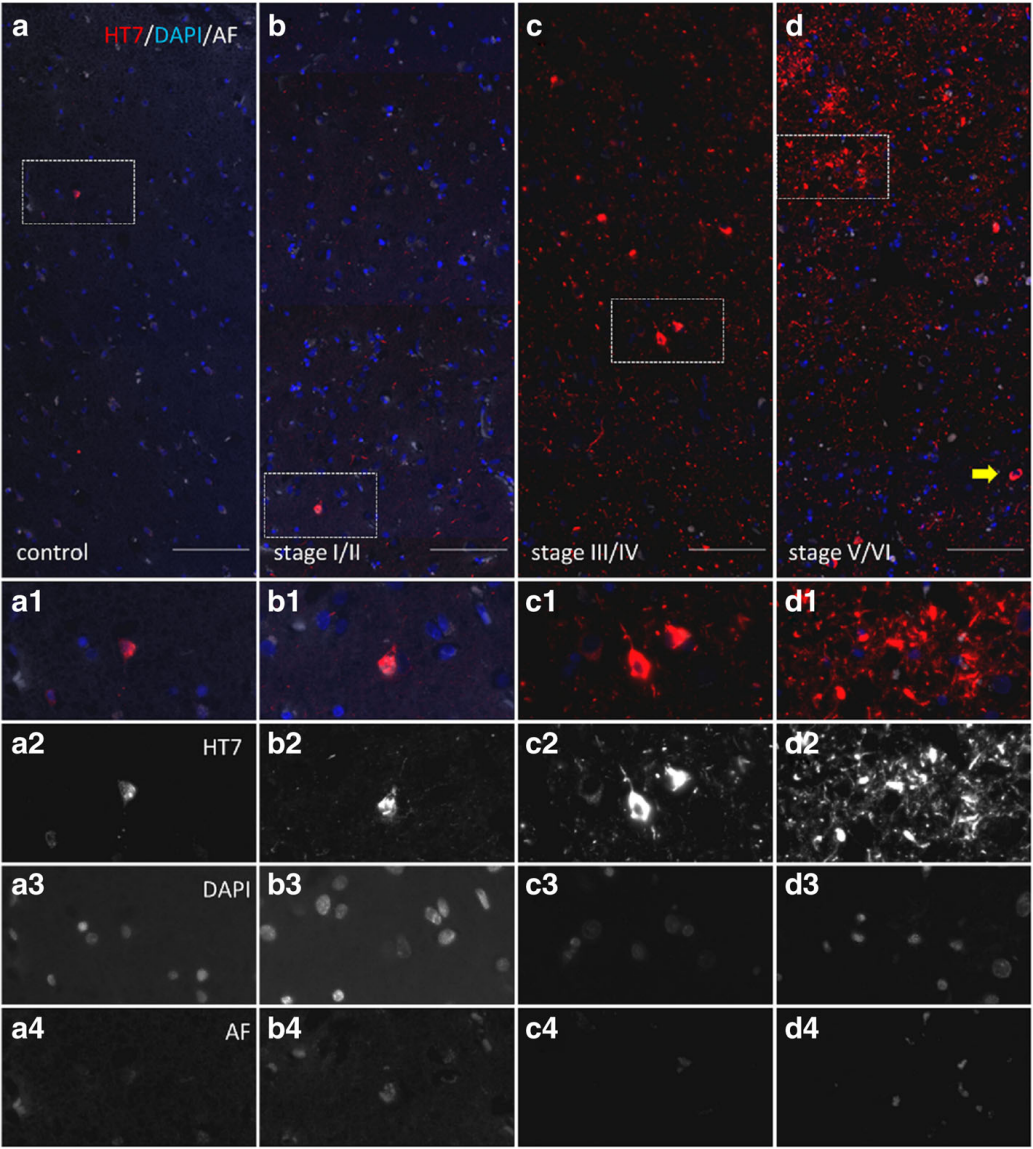
Representative images of total tau labeling in the transentorhinal cortex grey matter of AD cases. Labeling of total tau using HT7 antibody (a-d, a1-d1, a2-d2) and nuclei by DAPI staining (**a**-**d**, a1-d1,a3-d3). Autofluorescence is shown in white (**a**-**d**, a1-d1, a4-d4). Samples of healthy control tissue (a-a4, case 2), Braak stage I/II (b-b4, case 8), Braak stage III/IV (c-c4, case 11) and Braak stage V/VI (d-d4, case 16) are shown. Scale bar: 100 μm

**Fig. 8:**
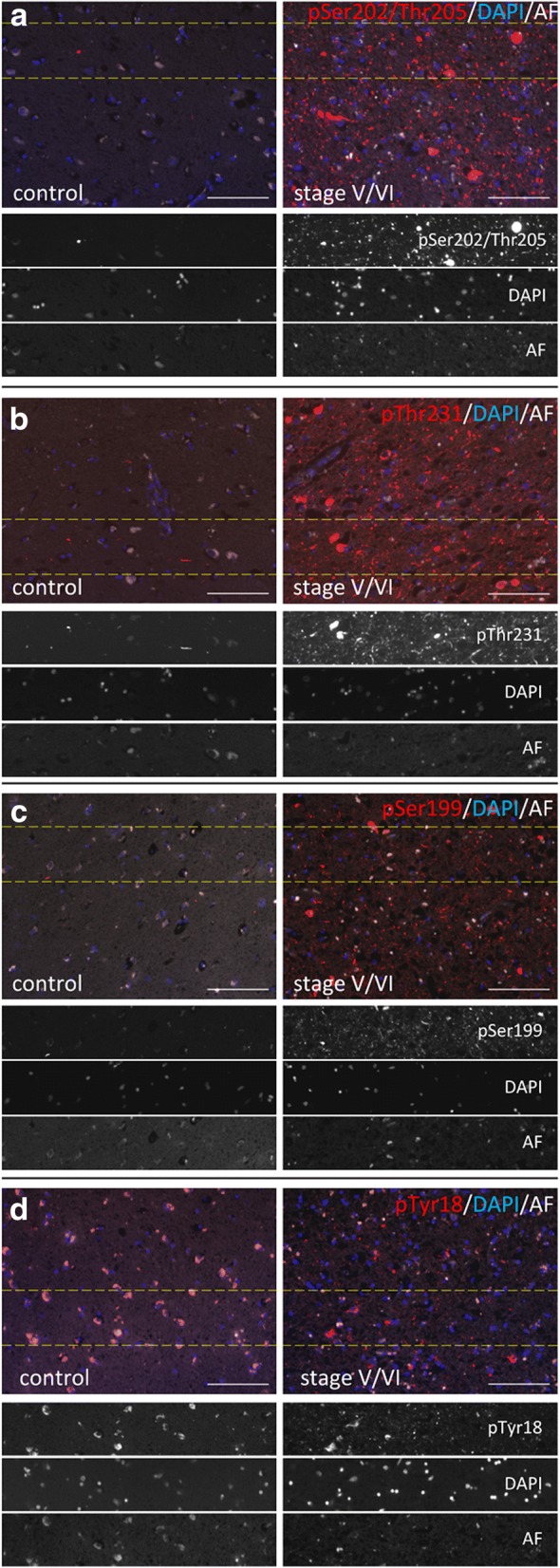
Representative images of ptau labelings in the transentorhinal cortex of control and AD cases. Labeling of pSer202/Thr205 (**a**), pThr231 (**b**), pSer199 (**c**), and pTyr18 (**d**) are shown. Tissues were additionally stained with DAPI to visualize nuclei. Autofluorescence is shown in white. Dotted lines indicate areas shown in grey scale images. Samples of healthy control tissue (case 2) and Braak stage V/VI (case 16) are shown. Scale bar: 100 μm

Representative images of total tau (Fig. 7) and pSer202/Thr205, pThr231, pSer199 and pTyr18 (Fig. 8) immunofluorescent labelings in the transentorhinal cortex of one case per Braak stage show the progressive increase of these ptau sites.

In summary, our quantitative analysis of AD brain tissue shows that a progressive increase of total tau and ptau expression can be observed in the transentorhinal region and in most of the analyzed isocortical regions. Staining of tissue with ThioS resulted only in a weak signal in the transentorhinal region and the occipital cortex. Labeling with antibodies against different tau phosphorylation sites showed that most ptau sites, namely pSer202/Thr205, pThr231, pSer199, pTyr18 and pSer422 were highly increased throughout the cortex at Braak stage V/VI. Only tau phosphorylation at Thr231 and Thy18 was already significantly increased at Braak stage III/IV. By contrast, pSer396 tau and pSer262 tau are only weakly expressed in all analyzed brain regions and only minor progression was observed. When comparing tau phosphorylation in percent relative to healthy controls, phosphorylation is specifically increased at tau Thr231, Ser199 and Tyr18. These data indicate that tau phosphorylation is a complex feature of AD progression, involving many but not all potential phosphorylation sites.

## Discussion

The current study was designed to analyze spatial patterns of tau phosphorylation at multiple residues in discrete anatomical regions during AD progression. Our data revealed a very similar phosphorylation profile of most of the analyzed ptau sites in the allo- and isocortex while expression levels of phosphorylated tau at Tyr18 and Thr231 was distinguishable between Braak stages. When normalized to controls, phosphorylation of tau at Tyr18, Ser231 and also Ser199 was much more increased at Braak stage V/VI compared to other residues, suggesting a relevance of these sites for AD progression and a crucial role in pathogenesis.

Since tau phosphorylation is a main characteristic of AD progression, several groups have already analyzed the temporal phosphorylation pattern of different tau sites by histological methods. According to Luna-Munoz and colleagues the phosphorylation of Thr231 tau is an early event in the neuronal pathology of AD [27]. The temporal analysis of pSer202/Thr205 tau and pSer396 tau is very controversial. Simic and co-workers found higher phosphorylation of Ser396 and Ser202/Thr205 tau in mild cognitive impairment (MCI) cases [38] suggesting a parallel phosphorylation of both residues. Temporal phosphorylation analyses by two other groups contradict these results, while one group found an earlier phosphorylation of pSer202/Thr205 tau [39], the other reported an earlier phosphorylation of pSer396 tau [30]. Zhou and colleagues performed dot blots and ELISA analyses of a whole series of ptau sites of AD medial temporal cortex samples and found mostly a similar temporal phosphorylation pattern as shown here for the entorhinal cortex. Tau phosphorylation of Ser396 for example, was a late event and only measurable in Braak stage V and VI [46]. Dot blot analyses of AD lateral temporal lobe samples by another group showed that tau phosphorylation at Ser202/205 and Ser396 simultaneously increases with increasing Braak staging, but also that they observe the increase already in Braak stage III/IV compared to results shown here or by Zhou and colleagues [20, 46]. By quantitatively analyzing ptau sites in different brain regions of AD cases we found that most analyzed ptau sites, pSer202/Thr205, pThr231, pSer199, pTyr18 and pSer422, have a very similar allo- and isocortical phosphorylation profile, suggesting that pSer202/Thr205 tau analysis by AT8 antibody could be replaced by any of these ptau sites when using this quantitative histological approach. For neuropathological assessment of Braak stages ptau Ser202/Thr205 immunopositivity is scored semi-quantitatively and is required to be mild (+) in the transentorhinal cortex to assign Braak stage I, while for stages higher than Braak stage I, severity of ptau Ser202/Thr205 immunopositivity in the transentorhinal cortex is not decisive as other areas, e.g. entorhinal cortex, occipitotemporal gyrus, middle temporal gyrus, occipital cortex need to show at least moderate (++) severity of immunopositivity [1]. Therefore our findings of a lack of a significant increase of ptau Ser202/Thr205 immunopositivity in the transentorhinal cortex between Braak stages 0 to IV is not at variance with neuropathological Braak staging. However, pSer202/Thr205 immunopositivity does indeed increase continuously in the transentorhinal cortex with increasing Braak stages. In our study the difference is only statistically significant between Braak stages 0-IV and V/VI. The phosphorylation of the analyzed tau sites starts in the isocortical regions late at Braak stage V/VI and consequently the analysis of the allocortical transentorhinal region is preferable. Indeed, the quantitative analysis of pTyr18 tau and pThr231 tau in the transentorhinal region shows a distinct progression between Braak stages that might be caused by highly increased phosphorylation relative to control tissue that could also be observed for pSer199 tau. Since phosphorylation of tau at residues Tyr18 and Thr231 occurs earlier compared to other sites, these phosphorylation sites might trigger disease progression and thus would represent an early therapeutic target.

Western blot analyses of total tau and the most prominent phosphorylation residues pSer202/Thr205, pThr231, pSer199, and pTyr18 overall validated the results obtained by immunofluorescent labeling. It has to be mentioned that tissue of not all human cases was available for blotting, so quantification and statistical analyses should be understood as preliminary. Blots for pSer202//Thr205 and pThr231 are not directly comparable, since different antibodies as used for immunofluorescent labelings had to be used for methodical reasons.

AD is known to affect women with a higher prevalence, which is among other reasons caused by hormonal differences. The higher frequency of AD cases in women is reflected in this study by the fact that only tissue of women was provided for Braak stage V/VI, while tissue of earlier Braak stages was predominantly from men. Although several in vitro and in vivo studies in rodents suggest an impact of sex hormones (e.g. estrogens, progesterone and prolactin) and therefore gender on tau phosphorylation, there are so far no studies in AD patients validating these results in humans (for review see [33]). Future analyses of *post mortem* AD tissue and in vivo imaging of AD patients should shed light on the impact of gender on tau phosphorylation.

In addition to the severe increases in tau phosphorylation also the ThioS signal, that includes amyloid plaques, was highly increased in several allo- and isocortical regions. The ThioS signal increase partially already started at Braak stage III/IV and thus earlier than the increase of ptau. It has been shown that ptau residues, Tyr18, Thr231 and Ser199 can be phosphorylated by Aβ via different kinases like Fyn [18, 35, 37, 42, 44, 45], GSK-3β [3, 23, 24] or CDK5 [6, 25]. The activation of tau by Aβ is further demonstrated to be involved in the early formation of neurofibrillary tangles, synaptic loss, neurodegeneration as well as cognitive deficits [2, 3, 11, 25] and thus in the development of the most prominent AD pathologies. These results were derived from AD cell and animal models but our study might suggest a similar effect of Aβ on tau phosphorylation at residues Tyr18, Thr231 and Ser199 in the human disease. Further analyses are needed to validate this hypothesis in humans. A valid tool to analyze such events in vivo might be the use of Pittsburgh compound B (PiB) analysis combined with tau tracer that are currently under development [5, 17, 19, 36].

Isocortical tau pathology is only sparse at early Braak stages I to IV and hence use of higher magnification and modified image analysis parameters may be necessary to reveal subtle changes in isocortical tau pathology at early Braak stages. Additionally, co-labeling of different markers to quantify ptau only in distinct areas or cell populations combined with an increased number of investigated brain regions may be helpful to gain a better understanding of earliest isocortical tau pathology. A tissue microarray method that was developed recently allows to examine over 35 brain regions on one slide could be used for such purposes [43]. However, our study investigated only five brain regions in a limited number of cases and represents therefore proof of principle. Our data need to be confirmed by including more cases and assessing a higher number of brain areas.

## Conclusion

We show here for the first time the phosphorylation profile of different tau sites in allocortical and isocortical regions of human brains with tau pathology ranging from Braak stages 0 to VI using quantitative rater-independent immunofluorescent labeling. Our data suggest that the profile of ptau in the isocortex is comparable between all of the analyzed ptau sites while expression levels of ptau at Thr231 and Tyr18 in the transentorhinal region are distinguishable between Braak stages combined with the highest tau phosphorylation relative to controls at these sites and Ser199.

## Additional file

Additional file 1: Online Source 9 Double labeling of pSer262 and pSer202/Thr205 tau in the temporal cortex at Braak stage V/VI. Images show different labeling pattern of pSer262 (arrows) and pS202 (white arrowheads) as well as their overlay (yellow arrowheads) (a1) and single fluorescence images (a2,3,4) of case 17. AF: autofluorescence. Scale bar: 20 μm. Online Source 10 Example of measurement procedure of tau pSer262. Objects in the unlabeled autofluorescence channel were detected by thresholding (red in a1). The resulting mask images (a2) were then subtracted from tau pSer262 images to remove autofluorescence (a3). The resulting images were Edge+ filtered (a4) to facilitate threshold-based detection of tau pSer262-positive objects (red outline in a5). These outlines were then loaded onto the raw images to quantify original tau pSer262 signal (red outline in a6). AF: autofluorescence. Scale bar: 20 μm. Online Source 11 Example of detecting ThioS-positive amyloid-β but not NFTs. Image a displays the co-labeling of ThioS (green) and HT7 (red), while images b and c, respectively, show single channel images. ThioS shows intense labeling of plaque-associated β-sheets (b, asterisk) whereas tangles are only weakly labeled (c, arrows) (c). A combination of threshold-based identification of ThioS and size restriction (d‘, green rectangle) enables quantification of ThioS+ plaque labeling (red highlighted) but not tangles (d). ThioS: ThioflavinS. Scale bar: 20 μm. (PDF 599 kb)

## Abbreviations

AD: Alzheimer’s disease
ANOVA: Analysis of variance
CiCtx: Cingulate cortex
FrCtx: Frontal cortex
NFT: Neurofibrillary tangle
NT: Neuropil threads
OcCtx: Occipital cortex
ptau: Phosphorylated tau
TeCtx: Temporal cortex
TEntR: Transentorhinal region
