# Supplementary material for: Phosphorylation of different tau sites during progression of Alzheimer’s disease
Source: Acta Neuropathol Commun. 2018 Jun 29;6:52. doi: 10.1186/s40478-018-0557-6 (PMC6027763; doi:10.1186/s40478-018-0557-6)
Supplement: Supplementary file 1 — Online Source 9 Double labeling of pSer262 and pSer202/Thr205 tau in the temporal cortex at Braak stage V/VI. Images show different labeling pattern of pSer262 (arrows) and pS202 (white arrowheads) as well as their overlay (yellow arrowheads) (a1) and single fluorescence images (a2,3,4) of case 17. AF: autofluorescence. Scale bar: 20 μm. Online Source 10 Example of measurement procedure of tau pSer262. Objects in the unlabeled autofluorescence channel were detected by thresholding (red in a1). The resulting mask images (a2) were then subtracted from tau pSer262 images to remove autofluorescence (a3). The resulting images were Edge+ filtered (a4) to facilitate threshold-based detection of tau pSer262-positive objects (red outline in a5). These outlines were then loaded onto the raw images to quantify original tau pSer262 signal (red outline in a6). AF: autofluorescence. Scale bar: 20 μm. Online Source 11 Example of detecting ThioS-positive amyloid-β but not NFTs. Image a displays the co-labeling of ThioS (green) and HT7 (red), while images b and c, respectively, show single channel images. ThioS shows intense labeling of plaque-associated β-sheets (b, asterisk) whereas tangles are only weakly labeled (c, arrows) (c). A combination of threshold-based identification of ThioS and size restriction (d‘, green rectangle) enables quantification of ThioS+ plaque labeling (red highlighted) but not tangles (d). ThioS: ThioflavinS. Scale bar: 20 μm. (PDF 599 kb) [file 40478_2018_557_MOESM1_ESM.pdf]

Phosphorylation of different tau sites during progression of Alzheimer´s disease

Acta Neuropathologica Communications

Joerg Neddens, Magdalena Temmel, Stefanie Flunkert, Bianca Kerschbaumer, Christina Höller, Tina Loeffler, Vera Niederkofler, Guenther Daum, Johannes Attems, Birgit Hutter-Paier

QPS Austria GmbH, Neuropharmacology, Parkring 12, 8074 Grambach, Austria, Birgit.hutter-paier@qps.com

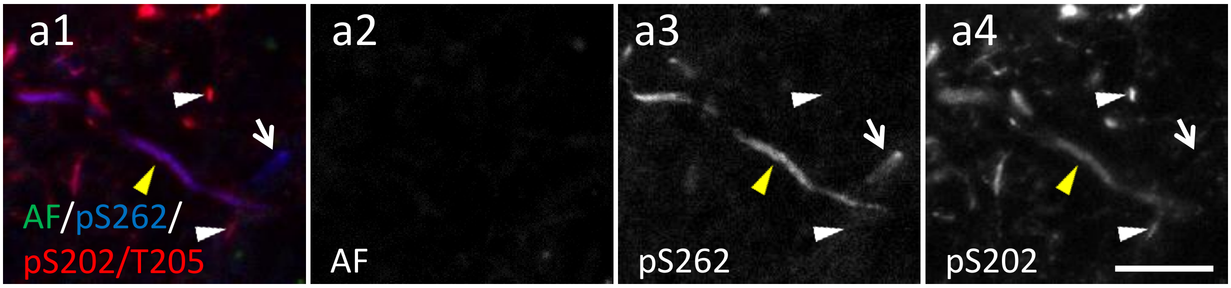

Online Source 9 Double labeling of pSer262 and pSer202/Thr205 tau in the temporal cortex at Braak stage V/VI. Images show different labeling pattern of pSer262 (arrows) and pS202 (white arrowheads) as well as their overlay (yellow arrowheads) (a1) and single fluorescence images (a2,3,4) of case 17. AF: autofluorescence. Scale bar: 20 µm.

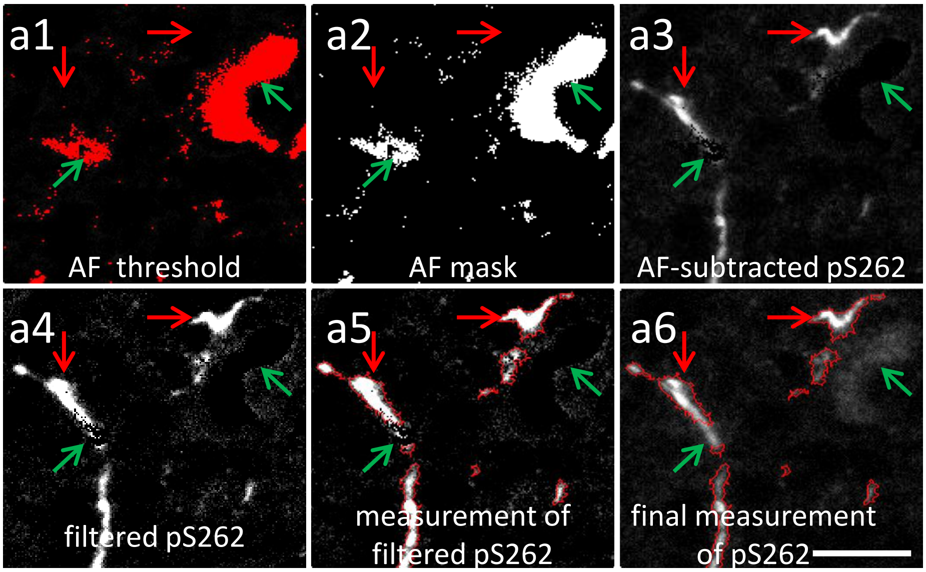

Online Source 10 Example of measurement procedure of tau pSer262. Objects in the unlabeled autofluorescence channel were detected by thresholding (red in a1). The resulting mask images (a2) were then subtracted from tau pSer262 images to remove autofluorescence (a3). The resulting images were Edge+ filtered (a4) to facilitate threshold-based detection of tau pSer262-positive objects (red outline in a5). These outlines were then loaded onto the raw images to quantify original tau pSer262 signal (red outline in a6). AF: autofluorescence. Scale bar: 20 µm.

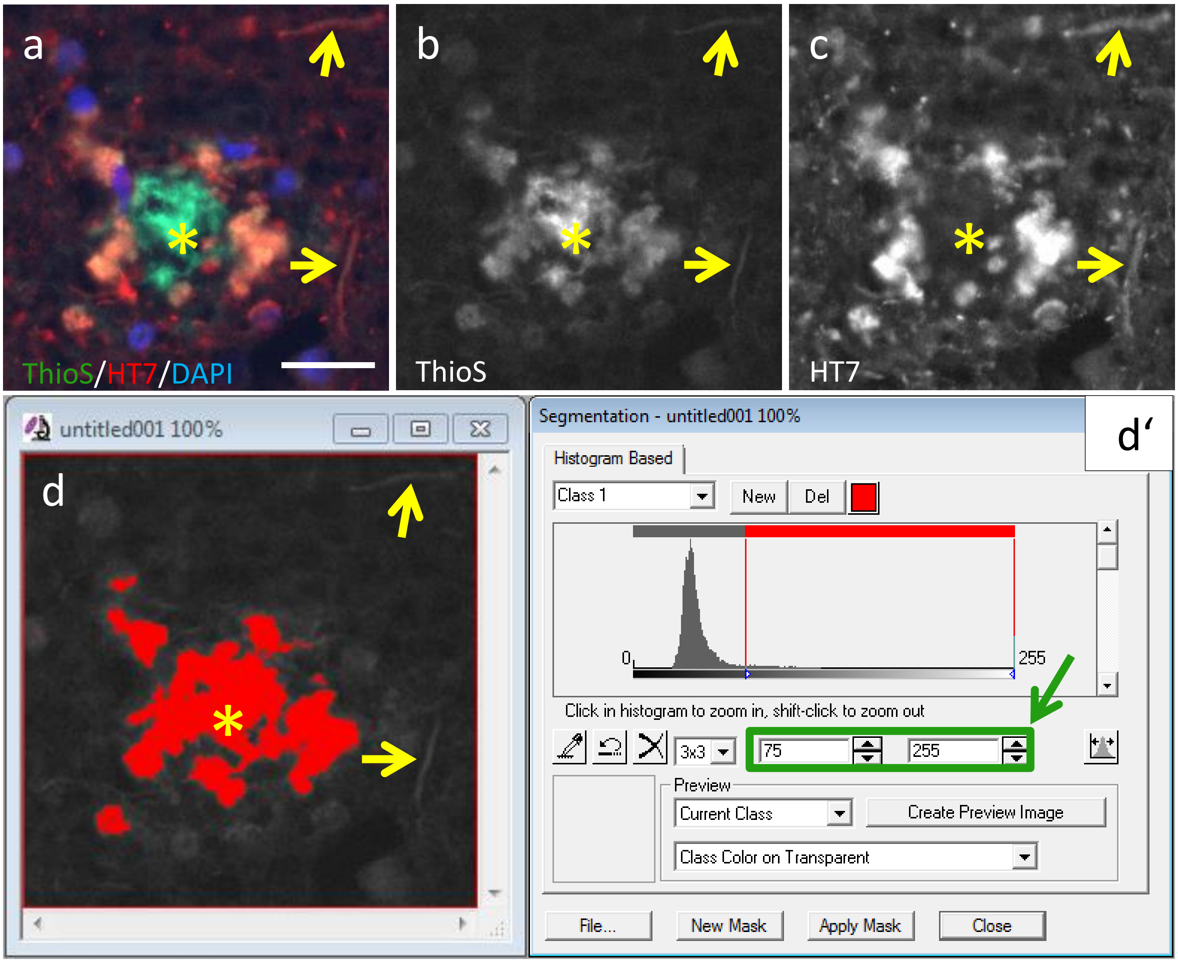

Online Source 11 Example of detecting ThioS-positive amyloid-β but not NFTs. Image a displays the co-labeling of ThioS (green) and HT7 (red), while images b and c, respectively, show single channel images. ThioS shows intense labeling of plaque-associated β-sheets (b, asterisk) whereas of tangles are only weakly labeled (c, arrows) (c). A combination of threshold-based identification of ThioS and size restriction (d‘, green rectangle) enables quantification of ThioS$^+$ plaque labeling (red highlighted) but not tangles (d). ThioS: ThioflavinS. Scale bar: 20 µm.
